# Novel approaches to estimate compliance with lockdown measures in the COVID-19 pandemic

**DOI:** 10.7189/jogh.10.010348

**Published:** 2020-06

**Authors:** Asiyah Sheikh, Zakariya Sheikh, Aziz Sheikh

**Affiliations:** 1 Medical student, University of Edinburgh, Edinburgh, UK; 2 Usher Institute, University of Edinburgh, Edinburgh, UK

A lockdown is a social distancing intervention that aims to minimise physical contact between individuals and groups in order to reduce transmission of a communicable disease [1]. Social distancing measures are typically introduced in an attempt to reduce and/or delay the peak of an epidemic/pandemic, to minimise the potential for surges in health care utilisation and to protect vulnerable groups. In the context of COVID-19, the World Health Organization has encouraged use of the term ‘physical distancing’ instead of social distancing to highlight that the aim of this intervention is only to reduce physical contact, not social contact which is often still possible through telephone and video calls, and social media [2]. There are a range of physical distancing measures, which can be broadly categorised as operating at the individual (eg, to support self-isolation of confirmed or suspected cases) or population levels (eg, closing of schools or workplaces) [1].

The lack of any effective pharmaceutical interventions for COVID-19 or vaccine against SARS-CoV-2 and the resulting failure to contain the virus has led to widespread adoption of physical distancing measures [3]. For example, according to the United Nations Educational, Scientific and Cultural Organization (UNESCO), 188 (96%) countries have by April 2020 implemented nationwide closure of schools [4]. Estimating compliance of policies encouraging physical distancing is crucial to assess their effectiveness and for accurate modelling of the future course of the pandemic. This viewpoint highlights three novel approaches that are now being deployed in some countries to assess compliance with physical distancing measures in the context of the COVID-19 pandemic. It is our hope that, by drawing attention to these measures, other parts of the world may also consider, where feasible and appropriate, deploying similar approaches.

## NOVEL APPROACHES TO ESTIMATING PHYSICAL DISTANCING AND COMPLIANCE WITH LOCKDOWN

### Global positioning system (GPS) data provided by mobile phone carriers

A number of governments have been provided anonymous aggregated mobile phone GPS data allowing monitoring of compliance with physical distancing at the population level (eg, Austria, Germany and Italy) [5]. In contrast, other countries are using more invasive techniques, using data on individuals, to assess compliance with physical distancing measures, trace contacts and enforce quarantine orders (eg, China, South Korea, Taiwan) [5].

### Mobile phone GPS data provided by other technology companies

A number of other technology companies (eg, IBM, Uber) are also sharing GPS data with academic institutions, governments and health providers (eg, Facebook Disease Prevention Maps have been shared with the London School of Hygiene & Tropical Medicine, which has played a major role in developing COVID-19 models for the UK) [6]. Recently, Google has published community mobility reports containing information on changes in mobility for six categories of location (eg, retail & recreation, parks and home) for 131 countries at national and local levels, which will be regularly updated [7]. The UK Government is using these data source as well as data on transport use to inform decision making, and have shared some of these in a recent press conference [8].

**Figure 1:**
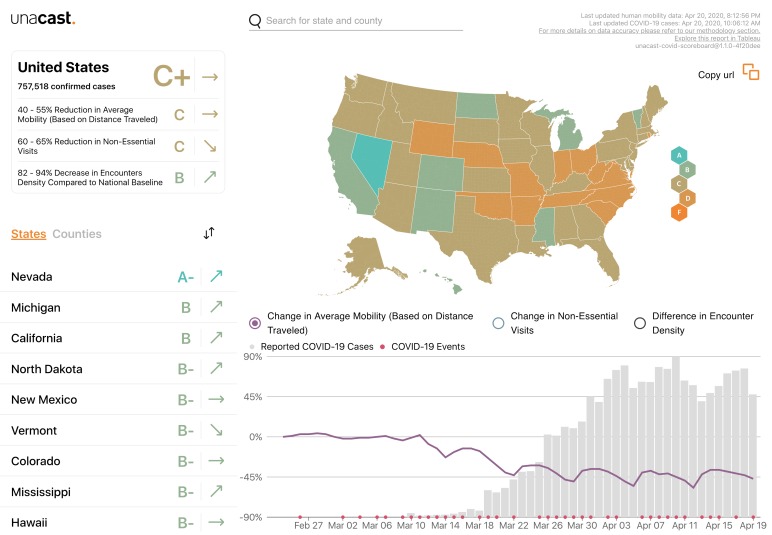
Screenshot of Unacast Social Distancing Scoreboard as of the 22^nd^ of April 2020 [9].

Similarly, Unacast has repurposed location and movement data that they collect via mobile phones to create a daily updated interactive scoreboard for “social distancing activity” for the United States that gives data at national, state and county levels (**Figure 1**) [9]. As this data is hyperlocal it allows comparisons of the effectiveness of physical distancing policies to be drawn, which can prove useful in the context of shaping regional and national public health messaging.

### Traffic congestion and public transport usage

**Figure 2:**
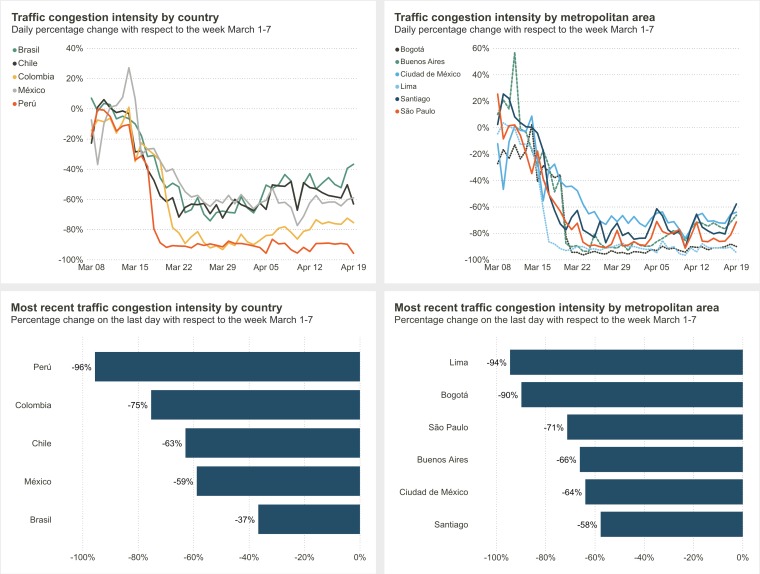
Screenshot of part of Inter-American Development Bank and IDB Invest Coronavirus Impact Dashboard as of the 22^nd^ April 2020 [10].

Alternative proxy measurements for physical distancing compliance include use of data on traffic congestion and public transport, which are often routinely collected through various means (eg, mobile phone GPS, road sensors). For example, the Inter-American Development Bank and IDB Invest have developed a public COVID-19 dashboard for Latin American and Caribbean countries including traffic data supplied by the navigation apps, Waze & Moovit (**Figure 2**) [10,11]. Similarly, CityMapper has created a mobility index by comparing typical and current usage of its navigation app [12].

**Figure:**
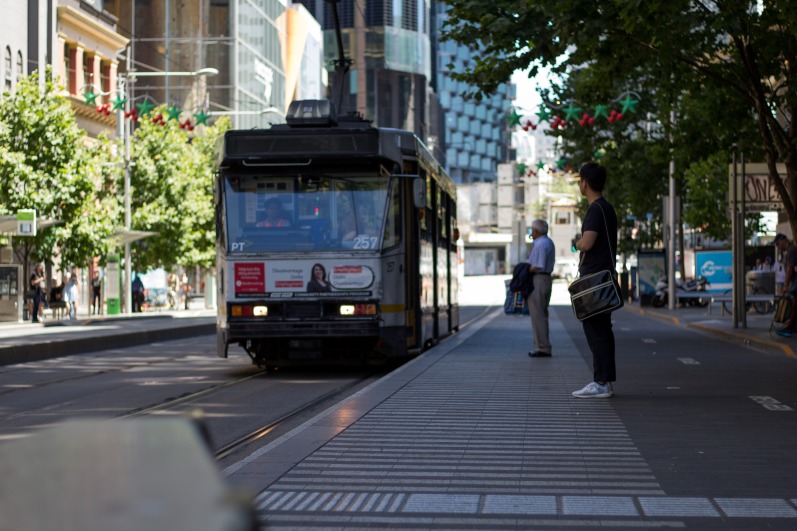
Photo: From the collection of Mateusz Glogowski (used with permission).

## CONCLUSIONS

COVID-19 has resulted in the introduction of lockdowns on an unprecedented scale in an attempt to modify the course of the pandemic and mitigate its effects. Measuring compliance with physical distancing is crucial to inform modelling deliberations and enable evidence-based policy making. We have summarised three key approaches that are currently being used to help estimate compliance with lockdowns in the context of the COVID-19 pandemic. Although currently used in a small minority of countries, there is substantial opportunity to scale up use of these approaches globally.
